# Nitrergic modulation of ion channel function in regulating neuronal excitability

**DOI:** 10.1080/19336950.2021.2002594

**Published:** 2021-11-22

**Authors:** Jereme G Spiers, Joern R Steinert

**Affiliations:** a Department of Biochemistry and Genetics, La Trobe Institute for Molecular Science, La Trobe University, Melbourne, Australia; b School of Life Sciences, Queen’s Medical Centre, University of Nottingham, Nottingham, UK

**Keywords:** Nitric oxide, excitability, ion channels, post-translational modification, neuron

## Abstract

Nitric oxide (NO) signaling in the brain provides a wide range of functional properties in response to neuronal activity. NO exerts its effects through different signaling pathways, namely, through the canonical soluble guanylyl cyclase-mediated cGMP production route and via post-translational protein modifications. The latter pathways comprise cysteine S-nitrosylation and 3-nitrotyrosination of distinct tyrosine residues. Many ion channels are targeted by one or more of these signaling routes, which leads to their functional regulation under physiological conditions or facilities their dysfunction leading to channelopathies in many pathologies. The resulting alterations in ion channel function changes neuronal excitability, synaptic transmission, and action potential propagation. Transient and activity-dependent NO production mediates reversible ion channel modifications via cGMP and S-nitrosylation signaling, whereas more pronounced and longer-term NO production during conditions of elevated oxidative stress leads to increasingly cumulative and irreversible protein 3-nitrotyrosination. The complexity of this regulation and vast variety of target ion channels and their associated functional alterations presents a challenging task in assessing and understanding the role of NO signaling in physiology and disease.

## Introduction

NO synthases (NOS) are widely expressed in various tissues of mammalian and non-mammalian species. Synthesis of NO and L-citrulline by NOS occurs via a two-step conversion process involving molecular oxygen (O_2_), L-arginine, and nicotinamide adenine dinucleotide phosphate (NADPH) as co-substrates. NO is produced via three different NOS isoforms, which are differentially expressed and differ in their calcium-dependence. Two isoforms, neuronal (nNOS, encoded by the *NOS1* gene) and endothelial (eNOS, encoded by the *NOS3* gene), are constitutively expressed and are calcium-dependent, whereas the inducible isoform (iNOS, encoded by the *NOS2* gene) is calcium independent and expressed in response to inflammatory stimuli. In the mammalian brain, all neurons express cytosolic nNOS to produce activity- and calcium-dependent NO and membrane-bound eNOS predominantly regulates vasodilation in the brain, though expression has also been demonstrated in dendritic spines [1]. Expression of iNOS, once induced in astrocytes and glia, contributes to nitrergic signaling during neuroinflammation in the brain and has been predominantly associated with neurodegeneration, which causes neurotoxicity affecting a large number of cellular signaling routes, including ion channel malfunctions [2–5].

The downstream effects of NO are diverse, concentration-dependent, and only partially reversible. Due to NO’s ability to diffuse, it represents a gasotransmitter and crosses membranes within a diffusion-limited space. Its physiological effects are relatively short lived, as it is oxidized to nitrite in the time frame of seconds being further metabolized. The effects of NO have been studied under numerous conditions where it affects presynaptic release mechanisms, synaptic plasticity, and neuronal excitability ([6–10]). The effects of NO are conserved across several species, including *Drosophila melanogaster* [11,12], Aplysia [13,14], and all mammalian species.

Within the mammalian hippocampus, NO exerts important regulatory functions related to learning and memory and synaptic plasticity, and NO’s ability to act as a volume transmitter has been well documented. Interestingly, nNOS expression is predominantly strong in hippocampal GABAergic non-principal neurons [15,16] with a relatively large subpopulation (approximately 30%) of hippocampal parvalbumin- and neuropeptide Y-positive GABAergic interneurons being nNOS positive [15,17]. Despite this limited and locally restricted NO production, NO can modulate postsynaptic and presynaptic functions of pyramidal neurons in an activity-dependent manner showing that the neuron producing NO and the neuron receiving NO signals differ in time and space. In addition, to regional and cellular differences in nNOS expression, functional NO release across hippocampal regions differs greatly [18] indicating the diversity of NO production and bioactivity.

**Figure 1.**
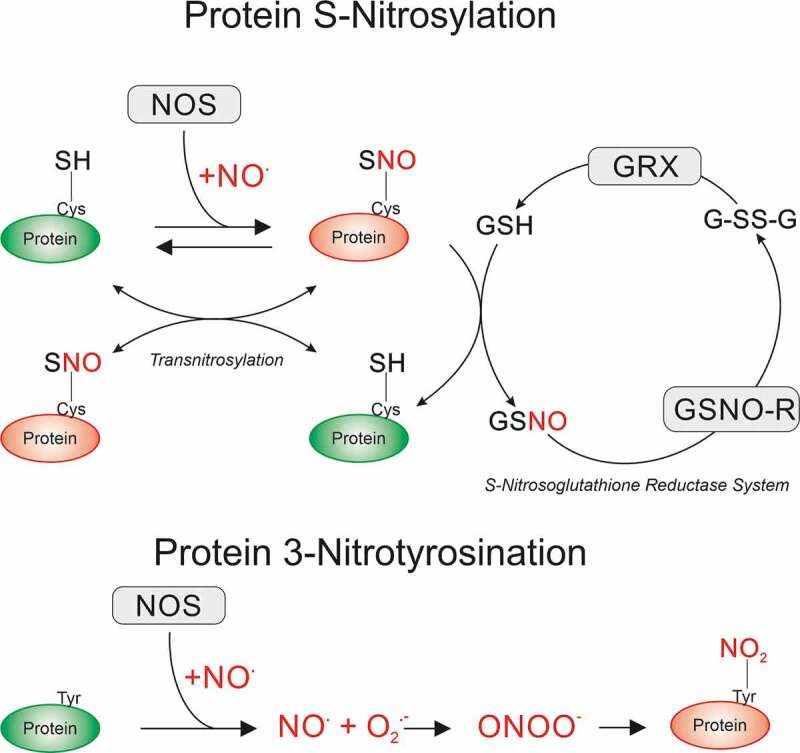
Post-translational modifications mediated by NO signaling. NO produced by nitric oxide synthases (NOS) can directly attack protein cysteine side chains to induce S-nitrosylation (SNO). This is a reversible modification that can be transferred to other protein cysteine side chains (transnitrosylation) or reduced by glutathione (GSH) to the native protein via the S-Nitrosoglutathione reductase (GSNO-R) system. The S-Nitrosoglutathione (GSNO) formed is then recycled via GSNO-R to oxidized GSH (GSSG) for further reduction to GSH by glutaredoxins (GRX). NOS-derived NO can also irreversibly attack tyrosine residues via the intermediate production of NO, superoxide radical (O_2_.^−^), and peroxynitrite (OONO^−^), forming 3-Nitrotyrosine modified proteins

The canonical NO-mediated activation of the soluble guanylyl cyclase and subsequent production of cyclic guanosine monophosphate (cGMP) to activate protein kinase G (PKG) is involved in protein phosphorylation. Additionally, NO-mediated post-translational modifications produce direct S-nitrosylation of protein thiols (addition of a nitrosyl ion NO^−^ to generate a nitrosothiol, S-N = O) [19] and protein tyrosine nitration (addition of NO_2_ to generate 3-nitrotyrosine, 3-NT, Figure 1). The latter pathway has been linked to iNOS-mediated NO generation with oxygen superoxide radical (O_2_^−^) presence facilitating the production of peroxynitrite (ONOO^−^) which occurs in several neuropathologies, general neuroinflammation, sepsis and ischemia/reperfusion [19,20]. Downstream NO signaling pathways depend on a multitude of factors and are affected by the degree of antioxidant activity, including the presence of transition metal complexes and the cellular redox status. Furthermore, reversibility of cGMP/PKG signaling by phosphatases or phosphodiesterase (PDEs), or S-nitrosylation via de-nitrosylation and trans-nitrosylation mediated by glutathione and S-nitroso-glutathione, may alter functional outcomes substantially.

When considering the actions of NO, it is important to be aware of regional, subcellular, and age-dependent fluctuations in addition to available concentrations of NO under physiological conditions. Most of the discussed data originate from studies using various chemical release donors, which possess different kinetics to generate NO [21]. Thus, exposure of a neuron to NO and the consequences for ion channel functions can differ drastically depending on which donor was applied. Importantly, there may be further physiological differences in NO release kinetics, depending on brain regions and developmental stages. In the hippocampus, there is a transient expression of nNOS in the pyramidal cell layer at P3-P7, which by P14 shows a reduced expression. At P21, there is a general lower expression, which is comparable to adulthood [22], suggesting that endogenous NMDAR-dependent NO production declines with age. In mice, we have previously shown that the size of NMDAR-mediated currents declined with age, reaching relatively comparable amplitudes between P14 and P21 (in the auditory brainstem) [23], again suggesting an age-dependent decline in NMADR-mediated NO generation.

The volume diffusion of NO can occur within a radius of several tens of μm [18,24] and at concentrations as low as fmolar to pmolar while lasting several seconds at these levels [18,25], indicating that volume transmitting creates a concentration gradient with varying outcomes in downstream signaling. The release profile of endogenously produced NO in response to NMDA perfusion into rat CA1 hippocampal regions shows a transient increase in NO levels between 100–350 nM (10–100 μM NMDA) [26,27]. Importantly, different NO release profiles between the hippocampal CA1/CA3/dentate gyrus regions have been detected following transient glutamate injection into hippocampal brain slices from 8–10 week old rats [18]. The amounts of released NO vary drastically between the regions, with peak levels reaching 3–4 fmoles·s^−1^ and lasting several tens of seconds. Other reports show NO levels persist for several tens of seconds at up to 300 pmoles following release in the cerebellum or hippocampus of P8-P10 old rats [28]. The use NO donors can generate a wide range of potentially active free NO levels. Most of the reviewed studies used NO donor concentrations of 5–200 µM but higher exposure levels of up to 5 mM were also reported. Depending on the donor this will lead to highly variable levels of free NO radicals, making a direct comparison between the observed effects on ion channels hard to interpret.

Although the importance of NO in the vasculature and immune response is widely accepted and well documented, this review will comprehensively evaluate the effects on neuronal properties mediated by NO regulation of different ion channel families, with focussed discussion of the most relevant ion channel types.

## Discussion: Nitrergic ion channel regulation

As neuronal communication fundamentally depends on action potential (AP) generation and propagation, which in turn requires the concerted actions of a variety of ion channels, NO is able to modulate information transmission by regulating those ion channels. The channels involved in determining AP thresholds, amplitudes, and waveforms comprise voltage-gated potassium channels (predominantly Kv1 and Kv7 – setting thresholds), the “delayed rectifier” Kv2 and Kv3 channels (responsible for AP repolarizations), sodium channels (voltage-activated: Na_v_ and persistent: Na_P_) as well as ion channels with more regulatory function such as large and small conductance calcium-activated potassium channels (BK, SK), ATP-regulated potassium channels (K_ATP_), hyperpolarisation-activated cyclic nucleotide-gated ion channels (HCN), or voltage-gated calcium channels (VGCC: L-type, N-type, P/Q-type, T-type, R-type).

***Kv1 channels***: Several subtypes of voltage-gated potassium channels are crucial for determining AP waveforms and AP firing thresholds. Low and high voltage-activated channels are setting thresholds and determine the repolarization phase of an AP. The low voltage-activated Kv1 channels are setting AP thresholds around resting membrane potentials in the range of −50 to −40 mV. NO has been shown to block Kv1 channels in squid giant fiber lobe neurons and when expressed in HEK 293 cells (Kv1.1 – Kv1.6), the currents were suppressed in a reversible manner by an NO donor with an IC_50_ of ~0.2 mM [29]. Similarly, NO (200 µM S-nitroso-N-acetylpenicillamine, SNAP; 100 µM sodium nitroprusside, SNP) reversibly blocks human Kv1.5 (hKv1.5) channels when expressed in mouse fibroblasts (Ltk^−^ cells) and importantly, the authors monitored NO bath concentration giving an IC_50_ of 340 nM NO. This block was mediated partially by a cGMP-dependent and S-nitrosylation-mediated mechanism with S-nitrosylation being proposed on Cys331 and Cys346 [30]. In addition, SNAP application inhibited native potassium currents in mouse ventricular myocytes elicited by Kv1.5 channels. In the human atrial myocardium, Kv1.5 is responsible for maintenance of the AP duration and height of the plateau phase which determines excitation-contraction coupling. As NO concentration in the myocardial tissue varies between 200–800 nM [31], under physiological conditions, NO likely maintains a “tonic” inhibition of human or mouse Kv1.5 channels, which in turn would be important for control of the atrial AP duration and height of the plateau phase.

***Kv2 channels***: High voltage-activated Kv2 (Kv2.1 and Kv2.2) channels are responsible for shaping AP waveforms and thus determining AP fidelity during firing trains of high activity. Predominantly expressed in the hippocampus, Kv2.1 currents are involved in setting excitabilities and neuronal firing during prolonged periods of AP firing. We have previously shown that Kv2.1 channels in hippocampal CA1 pyramidal neurons are reversibly blocked by an NO donor (10 µM NOC-5), a mechanism that was cGMP-independent and redox sensitive, again, suggesting S-nitrosylation as the main underlying mechanism [32]. This pronounced block (of ~50%) widens AP waveforms and leads to increased failure rates during prolonged periods of AP firing. Interestingly, another member of the Kv2 family, Kv2.2, is activated in the medial nucleus of the trapezoid body (MNTB) following prolonged periods of synaptic activity (>50 min), which requires NOS activation [33]. Activation of Kv2.1 channels in cortical neurons is implicated in apoptotic cell death, and increase in potassium efflux is associated with apoptosis. In this context, activation of the Kv2.1 channel has been shown after long-term (3 h) exposure of cortical neurons to 3-Morpholinosyndnomine (SIN-1), which decomposes in solution to release both NO and superoxide [34] leading to the formation of peroxynitrite. Treatment with 1 mM SIN-1 for 180 min led to a robust potentiation of potassium currents with relatively slow kinetics, consistent with that of Kv2 delayed rectifier channels [35]. Although not further investigated, this study suggests a peroxynitrite-mediated effect leading to channel activation, possibly due to 3-NT formation.

***Kv3 channels***: High voltage-activated Kv3 channels (Kv3.1 to Kv3.4) providing an important current for AP repolarization and, similarly to Kv2, they are responsible for shaping AP waveforms and maintaining high AP firing rates. Earlier studies showed that NO blocked Kv3.1 and Kv3.2 channels when expressed in CHO cells via the activation of the cGMP signaling cascade [36]. The effect of an NO donor on Kv3.1 and Kv3.2 currents is prevented by the phosphatase inhibitor okadaic acid and is mimicked by dialyzing of protein phosphatase 2A (PP2A) but not the related PP1^36^. Although the kinase itself and the sites at which it phosphorylates the channels are not yet known, this mechanism requires that the sites be basally phosphorylated in CHO cells. Consistent with these observations, we have previously shown that Kv3.1 channels are blocked by NO in a cGMP-dependent manner in principal neurons of the MNTB [33,37]. In these studies, the accumulation of NO was induced both in neurons receiving a direct presynaptic input as well as in adjacent neurons not receiving synaptic stimulation, indicating that NO acts as a non-synaptic volume transmitter in this nucleus. The major Kv3 family postsynaptic current in these neurons appears to be Kv3.1, the reduction of which in the MNTB prevents voltage-gated sodium channel recovery from inactivation and thus reduces firing frequencies, either during synaptically or depolarization-evoked postsynaptic activity.

***Kv4 channels (A-currents)***: Kv4 channels are fast inactivating potassium channels with subthreshold-activation responsible for dampening back-propagating APs and plateau potentials [38]. Although many of the voltage-gated potassium channel families exhibit varying degrees of voltage-dependencies of inactivation including Kv1.4, Kv3.3, Kv3.4, the family containing Kv4.1, Kv4.2 and Kv4.3 channels has classically been referred to as A-channels [39]. NO released by MAHMA-NONOate (100 µM) and DEA-NONOate (100 µM) produces a profound inhibition of Kv4.1 channels when expressed in *Xenopus* oocytes [40]. This inhibition was again redox sensitive and reversed by reduced glutathione, with data strongly suggesting that NO induces a di-sulfide bridge between Cys110 and Cys132 within the channel protein in intact cells. Although not further investigated in this study, inhibition of Kv4 would result in enhanced excitability and thus increases in spontaneous firing rates.

***Kv7 channels (M-currents)***: Kv7 channels are responsible for setting resting membrane potentials and thereby allowing other voltage-gated potassium and sodium channels to recover from inactivation. This non-inactivating potassium current with a threshold for activation below −60 mV [41] enables some M-channels to remain active at the resting membrane potential. Subsequently, opening of Kv7 channels leads to decreasing excitability of neurons thus providing essential functions for regulating neuronal activity. NO donor application (SNAP, IC_50_: 370 μM) suppresses M-currents, likely consisting of Kv7.2, Kv7.3 or Kv7.5 or a combination of the subunits, in nociceptive neurons resulting in increased neuronal excitabilities [42]. The same study further investigated the effects of NO on a specific subunit on the M-current, Kv7.4, when expressed in CHO cells. In these preparations, 1 mM SNAP inhibited Kv7.4 currents by ~30%, with ascorbate incubation reversing the inhibition to enhance the current and dithiothreitol (DTT) incubation substantially suppressing the current, suggesting a strong redox modification. This study further tested directly whether S-nitrosylation was the underlying reason for channel inhibition and confirmed that a triple cysteine at position 156–158 was the target for nitrergic modulation [42] supporting the conclusion that residues Cys156–158 are important for Kv7 channel modulation by NO. Conversely, in this study inhibition of nNOS by 1 mM L- Nω-Nitro-L-arginine (L-NNA) led to an increase in M-currents suggesting an endogenous regulation of the channel. These findings suggest an interesting possibility that M-currents may serve a role as cellular sensors conveying intracellular NO levels into changes of the electrical excitability of neuronal membranes.

***Large (BK, Maxi-K) and small (SK) conductance calcium-activated potassium channels***: Both channel types are involved in setting intrinsic excitabilities by adjusting resting membrane potentials in response to calcium availability. Inhibition of BK and SK channels will result in membrane depolarization and thus increased excitabilities. During AP firing, activation of these channels will shorten APs, accelerate repolarization and contribute to afterhyperpolarisation which results in an increased firing rate due to enhanced recovery of voltage-gated sodium channels from inactivation [43]. In B5 neurons from the buccal ganglion of *Helisoma trivolvis* NO donor application (NOC-7 and DEA-NONOate, 100 µM) and intrinsic production of NO leads to a suppression of SK currents [43,44], thereby depolarizing the membrane potential and enhancing excitability.

Regulation of BK channels by NO has been further shown to augment BK channel activity *via* the classical sGC/cGMP/PKG pathway in nerve terminals of the posterior pituitary [45]. This regulation has been studied more prominently in the vasculature smooth muscle in which PKG activation leads to phosphorylation of the BK channel at three specific PKG phosphorylation sites, an effect that results in enhanced channel activity [46]. Conversely, NO donors, SNP (100 µM) or DETA-NONOate (500 µM), increased activity of BK channels in rat CA1 pyramidal neurons which have previously undergone intermittent hypoxia. This activation was prevented by blockade of S-nitrosylation with N-ethylmaleimide (NEM, 1 mM) or 2-sulfonatoethyl methanethiosulfonate (1 mM) but not by inhibition of the cGMP pathway with ODQ or 8-bromo-cGMP [47]. Although the author no further investigated the role of BK current enhancement, these channels contribute to controlling the speed of AP repolarization which therefore impacts on neuronal excitability. Due to their presence in nerve terminals, they also regulate transmitter release. BK channels have been shown to be present in the dendrites of some neuronal populations where they can regulate the magnitude and duration of dendritic spikes [48]. In B5 neurons from the buccal ganglion of *Helisoma trivolvis* application of NO donors (NOC-7, DEA-NONOate, 100 µM) leads to a suppression of BK currents [44] contributing to membrane depolarization and increasing neuronal excitability.

***Voltage-gated sodium currents (Na_v_)***: Several subtypes of Na_v_ channels exist with slightly different properties in terms of activation and inactivation kinetics. The predominate consequences of Na_v_ activity, once activated at threshold, are apparent during the AP depolarization phase. Thus, by impacting channel conductance, activation or inactivation kinetics, NO modulation of Na_v_ affects APs, which will have profound effects on waveforms and firing rates. We have recently shown that NO (NOC-5, 10 µM) induces a reduction of Na_v_ currents in CA1 hippocampal pyramidal neurons requiring both cGMP-dependent and independent signaling components [32] as either blocking sGC activity by ODQ or suppression of redox signaling by glutathione prevents the observed nitrergic effects. Similar effects of NO (PAPA-NONOate, 5 mM) have been reported in dorsal root ganglion (DRG) neurons, where NO application led to a strong suppression of Na_v_ amplitudes via a cGMP independent mechanism, likely suggesting a post-translational channel protein S-nitrosylation [49].

***Persistent sodium channel (Na_P_)***: In B5 neurons from the buccal ganglion of *Helisoma trivolvis* intrinsic NO activates Na_P_ leading to a slight membrane depolarization and increased excitability [43]. In parallel, in cultured hippocampal rat CA1 neurons, a NO donor (SNAP, 100 µM) activated Na_P_ [50] suggesting a species independent and conserved mechanism. Further evidence for NO’s effects on Na_P_ is provided by a study in Kenyon cells isolated from cricket mushroom bodies in which the NO donor GSNO (10 µM) enhances the number of single channel openings in a cGMP and PKG-dependent manner, highlighting that NO acts as a modulator of both fast-inactivating and persistent sodium channels and that persistent sodium channels are constantly upregulated by the endogenous cGMP/PKG signaling cascade [51]. In contrast, in DRG neurons, NO suppresses Na_P_ currents in a cGMP-independent manner [49], further adding to the complex and diverse functions of NO signaling.

***HCN (I_h_ current)***: HCN 1–4 channels are classically activated by cyclic nucleotides, including cGMP and cAMP, which generate an inward current [termed hyperpolarization-activated current (I_h_)] at membrane potentials negative to approximately −50 mV. I_h_ helps setting the resting membrane potential and thereby influences neuronal excitability, synaptic integration, and other neuronal properties [52,53]. The mechanisms and importance of their regulation has been studied in multiple systems, including Aplysia [54], and in the mammalian brain, this channel has been described in some neuronal populations as a major regulator of neuronal intrinsic excitability [53]. Voltage dependence of HCN channels is regulated by intracellular signaling cascades, which contain cyclic nucleotides, phosphatidylinositol biphosphate (PIP_2_), and the auxiliary subunit tetratricopeptide-repeat containing Rab8b-interacting protein (TRIP8b). Numerous studies have provided evidence for nucleotide-activated I_h_ currents in several neuronal settings, with additional evidence suggesting NO-mediated and cGMP-independent regulation. In magnocellular neurosecretory cells (MNC) of the supraoptic nucleus, NO induces a cGMP-independent reduction in HCN currents (HCN 3 and HCN 4) by forming S-nitrosothiol complexes [55,56]. These findings shed new light on the mechanisms that control electrical excitability of MNCs via the nitrergic system and strengthen the importance of NO’s ability to control hydroelectrolyte homeostasis, providing further evidence for cGMP-independent regulation of HCN channels. This direct NO-mediated cysteine modification of HCN channels has been further studied in hypoglossal motoneurons (HMN). Interestingly, the potentiating effects of NO on I_h_ were detectable in the presence of ODQ and blocked by the cysteine-specific oxidant NEM, suggesting that NO affects hypoglossal motoneurons by S-nitrosylation-mediated activation of I_h_ [57]. The authors propose a probable S-nitrosylation site in HCN 1 to be at Cys531 in the COOH-terminal region and in HCN 2 at Cys341 in the cytoplasmic loop between S4 and S5. The contrasting findings on NO’s modulation of HCN channels may be the result of differential expression of HCN channels (HCN 3/4 in MNCs versus HCN 1/2 in HMNs) further illustrating the great diversity of nitrergic signaling. These apparent contradicting findings could result from differential modulation of cysteines, depending on their local environment within the protein. Corresponding S-nitrosylation positions in HCN 3 and 4 are at Cys494 and Cys662, respectively. Structurally, HCN 1 also appears to be intrinsically more organized, with higher helical structures than HCN 3/4 which may contribute to the different observations. When assessing potential S-nitrosylation sites within the proteins using a group-based prediction algorithm [58], all HCN 1–4 channel proteins possess additional cysteine residues which can potentially be S-nitrosylated thus providing a potential functional impact.

***Voltage-gated calcium channels (VGCC)***: VGCC are subdivided into different subtypes with L-type (Cav1), P/Q-type (Cav2.1), N-type (Cav2.2), T-type (Cav3) and R-type (Cav2.3) being activated during the depolarization phase of an AP with different subtypes exhibiting different activation and inactivation kinetics and thus contributing differentially to whole cell calcium currents. VGCC can be regulated by second messengers and G-proteins, through calcium dependent inactivation, and via facilitation by interaction with calmodulin associated with the C-terminal tail of the α1 subunit [59] in the case for P/Q type and L-type channels (for review [60]). However, NO mediates further differential effects on different channel subtypes, which also exhibits specie-dependent specificity. In the mouse MNTB, application of the NO donor SNP (100 µM) induces a potentiation of L- and P/Q-type calcium channels [61], whereas R- and N-type channels are unaffected. Interestingly, the signaling route by which NO modulates the channels also differed, with L-type activation being cGMP independent, whilst P/Q-type channel modulation requires cGMP signaling. However, L-type calcium channel amplitudes were reduced following NO application in a cGMP-dependent manner in frog and rat hair cells [55,56]. Other studies in rat hippocampal [62] and cortical neurons [59] showed NO-mediated increases in L-type calcium currents. N-type channels were suppressed by the canonical NO/cGMP pathway in neuroblastoma cells, whereas P/Q-type current amplitudes were augmented by NO in Baby Hamster Kidney fibroblast (BHK) cells [63,64]. In isolated rat retinal ganglion neurons, activation of G-protein coupled receptors (sst5) initiates the NO-mediated canonical cGMP/PKG cascade which leads to a reversible suppression of T-type calcium currents [65]. Further evidence for nitrergic effects on T-type channels comes from a study in neurons of the reticular thalamic nucleus in which application of GSNO (IC_50_: 1.2 mM) and SNAP (1 mM) rapidly and reversibly inhibit current amplitudes, an effect abolished by hemoglobin or NEM, but not by 8-Br-cGMP and ODQ, suggesting redox sensitive regulation [66]. The effects diminished the amplitude of low-threshold calcium spikes and frequency of spike firing under physiological conditions.

The effects of NO (GSNO, IC_50_: 110 µM) on T-currents in acutely dissociated DRG neurons and recombinant T-channel currents in human embryonic kidney (HEK) cells [67] were further investigated showing biphasic effects on DRG T-type currents, with a reversible transient increase in the amplitudes of currents followed by inhibition that was very slowly and incompletely reversible. The enhancing effect of GSNO on DRG T-type currents, but not the inhibitory effect, was eliminated if cells were incubated with the nonspecific metal chelator diethylenetriaminepentaacetic acid, suggesting different mechanisms for T-type current modulation. The inhibitory effect of GSNO on T-currents in DRG neurons was abolished by pre-treatment of cells with NEM but was not affected by co-applications of ODQ. These results suggested that the inhibitory effect of GSNO on DRG T-type currents is likely via direct S-nitrosylation and not via G-protein-dependent/cGMP/PKG signaling. Furthermore, in HEK cells stably transfected with human variants of T-type channel isoforms, GSNO similarly inhibited recombinant Ca_V_3.1, Ca_V_3.2, and Ca_V_3.3 currents. Further molecular studies using mutated channels in which four conserved cysteine residues in repeat I and II of the Ca_V_3.2 channels were replaced with alanine confirmed that these cysteines are critical for the inhibitory effects of GSNO on Ca_V_3.2 currents [67].

The complex responses of calcium channels to NO exposure illustrates the importance of this signaling route as downstream calcium signaling itself, and its modulation, will have different and opposing effects on cellular functions such as ion channel regulation (BK, SK), neurotransmitter release (vesicular release machinery) or gene expression through CREB (Calcium Response Element Binding protein) and CaRF (Calcium Response Factor) [68,69].

***ATP sensitive potassium channels (K_ATP_)***: K_ATP_ channels, widely present in metabolically active tissues, form inwardly rectifying potassium channels (Kir6.x) subunits (Kir6.1 or Kir6.2) [70]. As their opening is determined by the cytosolic ADP/ATP ratio, K_ATP_ channels act as metabolic sensors, linking cytosolic energetics with cellular functions in various tissues [71]. In the central and peripheral nervous system, widely distributed K_ATP_ channels [72,73] regulate neuronal excitability, neurotransmitter release, ligand-receptor effects, and cell survival during metabolic stress [74,75]. Although this channel has important neuronal functions, not many nitrergic effects have been reported. NO (SNAP, 100 µM) has been shown to activate K_ATP_ channels in large rat DRG neurons in cell-free patches. These effects were reversed by the thiol reducing agent, DTT, prevented by the thiol-alkylating agent, NEM, and were independent of cGMP signaling. These findings indicate that the mechanisms by which NO activates K_ATP_ channels involve direct S-nitrosylation of cysteine residues within the regulatory subunit 1 [76]. However, indirect activation of K_ATP_ channels in DRG neurons by NO acting via the classical sGC/cGMP/PKG pathway has also been suggested in previous studies [77,78].

***NMDA receptors (NMDAR)***: NMDARs are major excitatory ionotropic glutamate receptors found predominantly on the postsynaptic membrane. Their activation depends on ligand binding as well as on a voltage-dependent magnesium block – known as co-incidence detection – and their regulation has important implications for synaptic plasticity mechanisms such as being involved in learning and memory or long-term potentiation [79]. Early studies provide evidence for S-nitrosylation of NMDAR subunit NR2A at Cys399, which negatively modulates channel activity under physiological conditions [80]. There is further evidence that endogenous NOS activity is able to inhibit NMDAR activity in primary neurons, supporting the observation that neurotransmission across synapses expressing NR2A-containing NMDAR can be inhibited by NO [80]. In addition to NR2A, the NR1 subunit of NMDARs can be S-nitrosylated at Cys744 and Cys798 and higher S-nitrosylation is observed at these sites under hypoxic conditions, limiting further NMDAR-induced excitotoxicity [81]. Under resting conditions, hippocampal nNOS is highly S-nitrosylated, a modification known to decrease enzyme activity. This S-nitrosylation-dependent suppression of nNOS activity, in conjunction with reduced NMDAR activity, presents a negative feedback loop to reduce calcium entry and NO production. During cerebral ischemia-reperfusion, S-nitrosylation of nNOS in the rat hippocampus is reduced, via denitrosylation at Cys331 being induced by NMDAR-dependent calcium influx [82]. This finding indicates a mechanism by which calcium entry is mediating the reversal of nNOS inhibition during ischemia-reperfusion. Similarly, both NMDA exposure and in vivo focal cerebral ischemia S-nitrosylate the tyrosine phosphatase, SHP-2, reducing its activity and attenuating downstream neuroprotective ERK1/2 pathway activation [83,84].

***AMPA receptors (AMPAR)***: AMPAR activation produces a major glutamatergic excitation of the postsynaptic neuron, facilitating depolarization and AP initiation. Calcium influx through activated AMPARs increased nNOS-derived NO, which in turn S-nitrosylates N-ethylmaleimide-sensitive factor (NSF) and augments its binding to the AMPAR GluR2 subunit, thereby modulating AMPAR recycling to promote surface expression [85]. This has recently been shown to be the result of enhanced S-nitrosylation of thorase, which promotes internalization of the AMPAR-thorase complex in addition to trans-nitrosylating NSF, ultimately modulating AMPAR membrane insertion [86]. Thus, both NSF and thorase S-nitrosylation impact on synaptic plasticity leading to impaired long-term potentiation (LTP) and depression (LTD). More direct NO effects have been shown for NMDAR-mediated NO production, which directly S-nitrosylates the AMPAR GluA1 regulatory subunit at Cys875, which enhances Ser831 phosphorylation resulting in enhanced AMPAR conductance, and in turn results in receptor endocytosis by increasing receptor binding to the AP2 protein of the endocytotic machinery [87]. This pathway presents another negative feedback loop to reduce calcium entry and NO production, similar to the one reported for NMDAR signaling.

***GABA receptors (GABAR)***: Ionotropic GABA_A_ receptors produce inhibitory synaptic responses once activated, and while previous studies have reported effects of NO on GABA release mechanisms (for review [6]), nitrergic effects on GABAR themselves are not widely reported. More recent studies show that the interaction of the inhibitory synaptic scaffolding protein, gephyrin, with nNOS in primary hippocampal neurons promotes gephyrin S-nitrosylation, reducing postsynaptic clustering of GABA_A_R and regulating cell surface expression of GABA_A_R [88,89]. NO (DEA-NONOate, 100 µM, GSNO, 1 mM) leads to S-nitrosylation of homomeric ρ1 GABA_C_ receptors when expressed in oocytes, which induces increased GABA responses. This modulation requires two cysteine residues within the extracellular loop (Cys177 and Cys191), whereas a third cysteine at the intracellular loop (Cys364) seems redundant for the observed nitrergic effects [90]. Previous studies have suggested that the extracellular Cys-loop in ρ1 subunits spontaneously fluctuate between oxidized (di-sulfide bond) and reduced (free sulfhydryls) states, with exposure to certain redox thiol agents influencing the equilibrium between these [90]. Thus, in an analogous manner, NO can react producing an S-nitrosylation of thiol groups at Cys-loop Cys177 and Cys191, and in turn, this covalent modification induces protein structural rearrangements that impact GABA binding and channel gating [90]. With limited data available, it is hard to draw firm conclusions about nitrergic action on physiological GABAR modulation, and more studies are required to identify new target signaling routes.

## Summary

**Figure 2.**
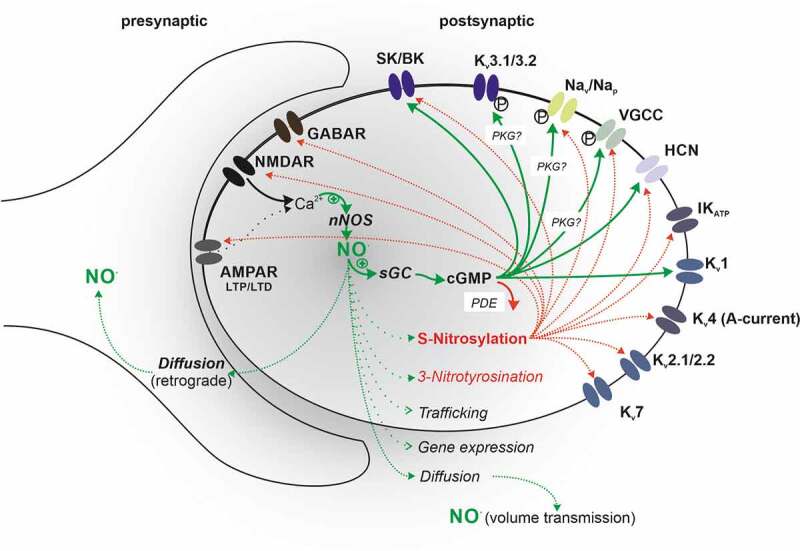
Schematic overview of ion channel modulation by S-nitrosylation and cGMP-dependent mechanisms. Several ion channel families are modulated by redox-sensitive mechanisms including S-nitrosylation. Other channel families are modified by both redox-sensitive and direct cGMP-dependent signaling (in addition to protein kinase G (PKG) activation with subsequent protein phosphorylation). A combination of the above signaling and modulation of different or multiple residues within one channel protein may lead to graded or opposing effects on channel conductances or kinetics with diverse outcomes for neuronal excitabilities. These complex modulatory effects depend on the redox state on the cell, amount of available NO and activities of mechanisms, which reverse the signaling, such as de-/trans-nitrosylation, breakdown of cGMP via phosphodiesterases (PDE), or dephosphorylation by phosphatases

Given the complexity of NO signaling that influences a multitude of pathways (including the canonical cGMP/PKG signalling [phosphorylation, direct cGMP effects], S-nitrosylation, and 3-nitrotyrosination) and mechanisms of their reversibility, it is not surprising that the literature presents overwhelming and sometimes contradictory findings related to NO signaling and neuronal function (Figure 2). The above-mentioned signaling routes produce effects on various ion channels, which, dependent on their activities, are involved in regulating intrinsic excitabilities, resulting in even more complex outcome for the given neuron (Table 1). Many reported studies suffer from not fully controlled experimental conditions, be it a defined redox status, controlled cGMP levels, controlled phosphatase or phosphodiesterase activities, defined intracellular ATP or calcium levels or even undefined NO release profiles as different donors used in numerous studies can generate distinct and varying levels of NO [21]. However, conducting further studies under refined conditions such as known time and space-dependent concentration profiles of NO release is required to uncover further functional properties of this signaling molecule at the level of individual neurons and across neuronal networks.

**Table 1.** Nitrergic modulation of ion channels

Channel	Nature of modulation by NO	Impact on cellular response	Ref
Kv1			
Kv1.1– 1.6	Current suppression via cGMP-dependent and S-nitrosylation dependent mechanisms	Membrane depolarization, increased excitability	[29,30]
Kv2			
Kv2.1	Current suppression in hippocampal CA1 pyramidal neurons via S-nitrosylation	AP widening and reduced firing rates during trains of activity	[32]
Kv2.2	Enhanced currents following long-term NO exposure in principal neurons of the mouse MNTB	Increases firing fidelity allowing high frequency AP trains	[33]
Kv3			
Kv3.1/3.2	cGMP-dependent block of currents in CHO cells (Kv3.1/3.2) and mouse MNTB principal neurons (Kv3.1)	Prolonged AP waveforms and reduced AP firing in MNTB neurons	[33,36,37]
Kv4/A-type	Redox-sensitive current suppression via cysteine S-nitrosylation	Increase in neuronal excitability with enhanced spontaneous activity	[40]
Kv7/M-current	Suppression of currents, inhibition of endogenous NOS activity enhances currents	Current inhibition leads to increased excitability in nociceptive neurons	[42]
SK channel	Intrinsic NO production suppresses SK currents in B5 neurons from the buccal ganglion	Leads to membrane depolarization enhanced excitability	[43,44]
BK channel	Current enhancement in CA1 pyramidal hippocampal neurons via S-nitrosylation and in smooth muscle via cGMP/PKG-mediated phosphorylation, Current inhibition in B5 neurons from the buccal ganglion	Enhanced AP repolarization in neurons and muscles	[44,46,47]
Voltage-gated Na^+^ channel	Suppression of current via a cGMP-dependent and a redox-sensitive component in hippocampal CA1 pyramidal and DRG neurons	Reduced rising phase of single AP depolarization and amplitudes resulting in lower firing frequencies due to limited channel availability	[32,49]
Persistent Na^+^ channel	Activation of currents in B5 neurons of the buccal ganglion of *Helisoma trivolvis*, in cultured hippocampal CA1 neurons and Kenyon cells isolated from cricket mushroom bodies; Current suppression in DRG neurons in a cGMP-independent manner	Induces membrane depolarization and increases excitability; Leading to membrane hyperpolarisation to reduce excitability	[43,49,51]
HCN (I_h_ current)	Activation of currents via cGMP signaling in many neuronal populations; cGMP-independent suppression of currents in magnocellular neurosecretory cells and hypoglossal motoneurons is mediated by S-nitrosylation	Reduction in membrane resistance suppresses the impact of synaptic currents on membrane potential changes; Reduces firing fidelity within trains of APs	[55–57]
VGCC			
L- type	Potentiation of currents in mouse principal MNTB neurons, rat hippocampal and cortical neurons; cGMP-dependent current suppression in frog and rat hairs cells	Enhanced calcium influx; Reduced calcium influx	[55,56,59,61,62]
P/Q-type	Potentiation of currents via cGMP signaling in mouse principal MNTB neurons and BHK cells	Enhanced calcium influx	[61,64]
N-type	Current suppression mediated by cGMP signaling in neuroblastoma cells	Reduced calcium influx	[63]
T-Type	Current suppression in rat retinal ganglion neurons by cGMP/PKG signaling and in reticular thalamic nucleus and DRG neurons via a redox-sensitive mechanism (S-nitrosylation)	Reduced calcium influx leading to diminished amplitudes of low-threshold calcium spikes and frequency of spike firing	[65–67]
ATP-sensitive K^+^ channels	Channel activation in DRG neuron cell-free patches, independent of cGMP signaling but redox-sensitive, cGMP-dependent activation in whole cell recordings from DRG neurons	Modulatory outcomes of K_ATP_ channel activation affect neuronal excitability, reduction of excitability in DRG and hippocampal pyramidal neurons	[76–78]
NMDAR	NR1 and NR2A subunit inhibition via S-nitrosylation	Reduction in calcium influx resulting in limited excitotoxicity	[80–82]
AMPAR	S-nitrosylation of N-ethylmaleimide-sensitive factor modulates AMPAR GluR2 surface expression, Direct GluA1 subunit S-nitrosylation	Enhanced GluR2 surface expression leads to stronger postsynaptic excitation; Increased AMPAR GluA1 conductance which facilitates its phosphorylation and reduced surface expression, thereby limiting overall receptor activities and impair LTP/LTD	[85–87]
GABAR	S-nitrosylation of gephyrin, modulates postsynaptic GABA_A_R clustering, Direct S-nitrosylation of two different Cys residues of GABA_C_R expressed in oocytes	Reduction of receptor clustering reduces inhibitory function of GABA signaling; Enhanced GABA responses increase inhibitory GABAergic function	[88–90]
